# Supplementary material for: Forward genetic screen of human transposase genomic rearrangements
Source: BMC Genomics. 2016 Aug 4;17:548. doi: 10.1186/s12864-016-2877-x (PMC4973553; doi:10.1186/s12864-016-2877-x)
Supplement: Additional file 1: Table S1. — Annotated DNA transposons in the HPRT1 gene. (DOC 32 kb) [file 12864_2016_2877_MOESM1_ESM.doc]

**Table S1. Annotated DNA transposons in the *HPRT1* gene**

| **Position** | **Transposon Name** | **Transposon Class** |
| --- | --- | --- |
| ChrX: 133609504-133609757 | MARNA | TcMar-Mariner |
| ChrX: 133612862-133613034 | MER104 | TcMar-Tc2 |
| ChrX: 133616475-133616651 | MER3 | hAT-Charlie |
| ChrX: 133623197-133623402 | Charlie7a | hAT-Charlie |
| ChrX: 133623432-133623581 | Eulor8 | TcMar |
| ChrX: 133624726-133624872 | MER5A | hAT-Charlie |
| ChrX: 133625413-133625487 | Tigger5 | TcMar-Tigger |
| ChrX: 133625475-133625553 | MER47C | TcMar-Tigger |
| ChrX: 133626916-133626974 | MER5A | hAT-Charlie |
| ChrX: 133628672-133628708 | MADE1 | TcMar-Mariner |
| ChrX: 133628926-133629162 | MER106B | hAT-Charlie |
| ChrX: 133630729-133630913 | MER5B | hAT-Charlie |
