# Supplementary material for: Forward genetic screen of human transposase genomic rearrangements
Source: BMC Genomics. 2016 Aug 4;17:548. doi: 10.1186/s12864-016-2877-x (PMC4973553; doi:10.1186/s12864-016-2877-x)
Supplement: Additional file 2: Table S2. — HPRT1 PCR primers and PCR amplicon locations. (DOC 33 kb) [file 12864_2016_2877_MOESM2_ESM.doc]

Table S2. HPRT1 PCR primers and PCR amplicon locations

| **#** | **ChrX location** | **Forward primer (5′-3′)** | **Reverse primer (5′-3′)** |
| --- | --- | --- | --- |
| 1 | [133594507-133599897](https://genome.ucsc.edu/cgi-bin/hgTracks?hgsid=470983985_h7QqI07AIlFcB6J2akoVFVOuBGJA&db=hg19&position=chrX:133594507-133599897&hgPcrResult=pack) | CCGGGTTCGGCTTTACGTC | CTGGAGGGGCCTAGAAGTGG |
| 2 | [133598482-133603643](https://genome.ucsc.edu/cgi-bin/hgTracks?hgsid=470983985_h7QqI07AIlFcB6J2akoVFVOuBGJA&db=hg19&position=chrX:133598482-133603643&hgPcrResult=pack) | ATGCCAACTAGCAATTTCTGCCCATTCC | GTTCAGTGCCTGGCTACAGGTTCTG |
| 3 | [133603619-133608070](https://genome.ucsc.edu/cgi-bin/hgTracks?hgsid=470983985_h7QqI07AIlFcB6J2akoVFVOuBGJA&db=hg19&position=chrX:133603619-133608070&hgPcrResult=pack) | CAGAACCTGTAGCCAGGCACTGAAC | GTATACATGCATAGCCAGTGCTTGAG |
| 4 | [133608045-133614449](https://genome.ucsc.edu/cgi-bin/hgTracks?hgsid=470983985_h7QqI07AIlFcB6J2akoVFVOuBGJA&db=hg19&position=chrX:133608045-133614449&hgPcrResult=pack) | CTCAAGCACTGGCTATGCATGTATAC | GAACCTTGACTGCAACTGAGGGAA |
| 5 | [133614426-133619418](https://genome.ucsc.edu/cgi-bin/hgTracks?hgsid=470983985_h7QqI07AIlFcB6J2akoVFVOuBGJA&db=hg19&position=chrX:133614426-133619418&hgPcrResult=pack) | TTCCCTCAGTTGCAGTCAAGGTTC | ACCCAGGCATGACATGAACCA |
| 6 | [133619398-133624673](https://genome.ucsc.edu/cgi-bin/hgTracks?hgsid=470983985_h7QqI07AIlFcB6J2akoVFVOuBGJA&db=hg19&position=chrX:133619398-133624673&hgPcrResult=pack) | TGGTTCATGTCATGCCTGGGT | ACTGCTTAGCCTCTCGGTGCTAAG |
| 7 | [133624650-133627625](https://genome.ucsc.edu/cgi-bin/hgTracks?hgsid=470983985_h7QqI07AIlFcB6J2akoVFVOuBGJA&db=hg19&position=chrX:133624650-133627625&hgPcrResult=pack) | CTTAGCACCGAGAGGCTAAGCAGT | CATACCTTGCGACCTTGACCATC |
| 8 | [133627603-133635224](https://genome.ucsc.edu/cgi-bin/hgTracks?hgsid=470983985_h7QqI07AIlFcB6J2akoVFVOuBGJA&db=hg19&position=chrX:133627603-133635224&hgPcrResult=pack) | GATGGTCAAGGTCGCAAGGTATG | CACGTGTAAGCTAGATGGCTCCTA |
